# Development of Mixed Micelles for Enhancing Fenretinide Apparent Solubility and Anticancer Activity Against Neuroblastoma Cells

**DOI:** 10.2174/0115672018333862240830072536

**Published:** 2024-09-03

**Authors:** Guendalina Zuccari, Alessia Zorzoli, Danilo Marimpietri, Silvana Alfei

**Affiliations:** 1 Department of Pharmacy, University of Genoa, Viale Benedetto XV 16132 Genoa , Italy;; 2 Stem Cell Laboratory and Cell Therapy Center, IRCCS Istituto Giannina Gaslini, via Gerolamo Gaslini 5, 16147 Genoa, Italy

**Keywords:** Fenretinide, neuroblastoma, mixed micelles, vitamin E TPGS, lipophilic drug, drug delivery systems

## Abstract

**Introduction/Objectives:**

The purpose of the study was to evaluate the suitability of mixed micelles prepared with D-α-tocopheryl polyethylene glycol succinate (TPGS) and 1,2-distearoyl-glycero-3-phosphoethanolamine-N-[methoxy(polyethyleneglycol)-2000] (DSPE-PEG) to encapsulate the poorly soluble anticancer drug fenretinide (4-HPR).

**Methods:**

After assaying the solubilization ability of the surfactants by the equilibrium method, the micelles were prepared using the solvent casting technique starting from different 4-HPR:TPGS: DSPE-PEG w/w ratios. The resulting formulations were investigated for their stability under storage conditions and upon dilution, modelling the reaching of physiological concentrations after intravenous administration. The characterization of micelles included the determination of DL%, EE %, particle size distribution, Z-potential, and thermal analysis by DSC. The cytotoxicity studies were performed on HTLA-230 and SK-N-BE-2C neuroblastoma cells by the MTT essay.

**Results:**

The colloidal dispersions showed a mean diameter of 12 nm, negative Zeta potential, and a narrow dimensional distribution. 4-HPR was formulated in the mixed micelles with an encapsulation efficiency of 88% and with an increment of the apparent solubility of 363-fold. The 4-HPR entrapment remained stable up to the surfactants’ concentration of 2.97E-05 M. The loaded micelles exhibited a slow-release behaviour, with about 28% of the drug released after 24 h. On the most resistant SK-N-BE-2C cells, the encapsulated 4-HPR was significantly more active than free 4-HPR in reducing cell viability.

**Conclusion:**

Loaded micelles demonstrated their suitability as a new adjuvant tool potentially useful for the treatment of neuroblastoma.

## 1 INTRODUCTION

Cancer is a leading cause of death around the world [1]. However, chemotherapy triggers the death of fast-dividing cells in both healthy and tumour tissue, thus, the balance between the effectiveness of the anticancer drugs and the patient's ability to tolerate their side effects has to be optimized [2]. Nanomedicines can exceed some of the limitations of traditional chemotherapy due to their ability to selectively target tumour tissue, overcome biological barriers, enhance drug-circulation time, control drug-release kinetic, and allow superior dose scheduling [3]. In general, nano-sized delivery systems should be formulated to avoid the use of toxic excipients that may contribute to the side effects associated with chemotherapeutics [4]. In this context, several attempts have been put forward to reduce hypersensitivity reactions associated with paclitaxel injectable forms containing polyoxyethylated castor oil [5]. Among the colloidal drug delivery systems gathered under the umbrella of nanomedicine, micelles have been extensively investigated for encapsulating lipophilic drugs owing to their controlled drug release, stability, and solubilizing efficiency [3]. Micelles consist of amphiphilic molecules able to self-assemble above their critical micellar concentration (CMC) in colloidal supramolecular aggregates. The hydrophilic moiety, usually polyethylene glycol (PEG), forms the shell of the micelles, whereas the hydrophobic moiety forms their *core* in which poorly soluble drugs can be solubilized [6]. Typically, micelles are endowed with a mean hydrodynamic diameter usually less than 50 nm [7]. This feature allows the micellar systems to easily extravasate by crossing pathological fenestrations present in the body’s vasculatures in acute and chronic inflammation, cancer, and wound healing [8]. It has been demonstrated that sub-50 nm drug formulations achieve deeper tumour tissue penetration and more efficient cancer cell internalization, as well as a higher cellular response [9]. Secondly, the micelles are more suitable for the scale-up of the manufacturing process [10] and have been extensively applied in cancer treatment [11]. Certainly, liposomes are difficult and expensive to manufacture, despite the guidelines provided by the regulatory authorities [12]. It is noteworthy the case of AmBisome®, whose supply is provided only by Gilead Sciences since the attempts to develop generic liposomal formulations of Amphotericin B failed to demonstrate the equivalence with the originator [13]. On the contrary, the manufacturing of generic micellar formulation is more reproducible and standardizable, as proven by the different generic formulations available on the market of the micellar Taxotere®, because of its easy preparation at a large scale [14]. Although the abovementioned advantages of micellar formulations, emulsifiers may be observed as potentially dangerous following parenteral administration [15] [16]. Therefore, for the selection of surfactants to be used, it is of fundamental importance to consider their biocompatibility and tolerability. Among amphiphilic structures, which are able to increase the solubilization rate of poorly soluble drugs, D-α-tocopheryl polyethylene glycol succinate (TPGS) has attracted much attention in the scientific community since it showed *per se* promising anticancer effects. Indeed, TPGS can reverse P-glycoprotein (P-gp) [17] and block human breast cancer cells (MCF-7) growth with IC50 values of 102 µM and 56 µM at 24 and 48h, respectively [18]. In another study, TPGS was reported to trigger pro-oxidative stimuli and apoptosis in neuroblastoma cells (SK-N-SH) [19]. From the technological perspective, the main advantages of TPGS include its use as an absorption enhancer, emulsifier, solubilizer, permeation enhancer, and stabilizer of poorly soluble drugs [20]. In this context, TPGS has already been employed to improve solubility and permeation in oral, transdermal, and topic delivery of paclitaxel, etoposide, curcumin, retinoic acid, and resveratrol [21-25]. However, since TPGS CMC is relatively high (10^-4^ M) [21], when parenteral administration is needed, TPGS should be associated with another surfactant, allowing it to reach CMC within the range of 10^-5^-10^-6^ M, thus preventing micelles easy dissociation in the plasma. As an antitumor drug to be encapsulated into a micellar delivery system, fenretinide or N-(4-hydroxyphenyl)-retinamide (4-HPR) was selected because it was affected by poor biopharmaceutical properties. 4-HPR is a synthetic derivative of retinoic acid, belonging to the Class IV of the Biopharmaceutical Classification System [26]. To date, 4-HPR has failed to reach the market due to the unsatisfactory results shown in clinical investigations, mostly ascribable to the hepatic first-pass effect and the drug's slow dissolution rate [26]. Based on these considerations, the parenteral route of administration was considered almost mandatory to overcome these unfavourable features [26]. Our aim was to develop a novel micellar formulation by encapsulating the drug into mixed micelles formed by 1,2-distearoyl-glycero-3-phosphoethanolamine-N-[methoxy(polyethyleneglycol)-2000] (DSPE-PEG) and TPGS. DSPE-PEG already proved to form micelles with extended plasma circulation half-life, small size, and effective accumulation in tumour tissues [27]. In our goals, a successful 4-HPR encapsulation into the micellar *core* would have provided an increment of drug apparent solubility by an easily and scalable technique using well-tolerated ingredients without harmful co-solvents. Subsequently, the micelles were characterized by thermal analysis, drug release, dynamic light scattering, and cytotoxic activity against neuroblastoma cells in order to evaluate their potential applications in cancer treatment.

## 2 MATERIALS AND METHODS

### 2.1 Materials

TPGS was a gift from PMC Isochem (Vert Le Petit, France), 1,2-distearoyl-glycero-3-phosphoethanolamine-N-[methoxy(polyethyleneglycol)-2000] (DSPE-PEG) was purchased from Avanti Polar Lipids, Inc. (Alabaster, AL), while 4-HPR and all reagents were purchased from Merk (Milan, Italy).

### 2.2 Solubility Studies

#### 2.2.1 Determination of Solubility Vitamin E Parameter

Furthermore, to evaluate if TPGS could be a good solubilizer for 4-HPR, a reported method to predict the solubility of drugs in tocopherols was performed [28]. This assay called the Solubility Vitamin E Parameter (SVE), is defined as the solubility in chloroform divided by the solubility in methanol expressed in mg/mL. A fixed amount of 4-HPR (20 mg) was added with increasing solvent aliquots (50 µL), and after every addition, it was kept to equilibrate for 24 h. The concentration of saturation that was achieved in the latter sample still showed the presence of undissolved material.

#### 2.2.2 Effect of Surfactants on 4-HPR Solubility

In order to investigate the ability of TPGS and DSPE-PEG to effectively encapsulate 4-HPR, the shake-flask method was performed. Briefly, a weighed 4-HPR amount (3 mg) was dispersed in 10 mL milli-Q water and added with different amounts of surfactants. Maintaining the DSPE-PEG:TPGS molar ratio 1:2, the tested ratios by weights (w:w:w) of 4-HPR:TPGS:DSPE-PEG were: 1:0.5:0.46, 1:5:4.6, 1:10:9.3, 1:20:18.3, 1:30:28, 1:40:36.6, 1:50:46.6, 1:60:66 (w/w). After 48 h at 37 °C under stirring, the suspensions were filtered using a 0.22 µm filter (Minisart RC Sartorius, Göttingen, Germany) to remove undissolved drug particles. Aliquots of each filtrate were diluted with methanol and analysed by UV-Vis spectrophotometer (Evolution 300, ThermoFisher Scientific, Segrate, Italy) at λmax = 364 nm. All the samples were in triplicate, and the results were reported as the mean ± standard deviation (SD). TPGS: DSPE-PEG solutions alone were used as blank. The total 4-HPR solubility was calculated using a previously constructed standard calibration curve. The mean of three independent measurements was used to build the calibration graph.

### 2.3 Preparation of 4-HPR Mixed Micelles

Loaded mixed micelles were prepared using the solvent casting method. Briefly, a fixed amount of 4-HPR (3 mg) and an increasing amount of TPGS: DSPE-PEG (2:1 molar ratio) were solubilized in 6 mL of chloroform. The organic solvent was removed by rotary evaporation at 40°C to form a homogeneous thin film. After cooling to room temperature, the thin layer was hydrated with 10 mL HEPES-buffered saline (HBS, 10 mM, pH 7.4) and resuspended at room temperature by gentle shaking for 4 h. Unincorporated 4-HPR was separated by filtering the micelle suspension through a 0.22 µm filter (Minisart RC Sartorius, Göttingen, Germany). The void micelles were prepared as described above without adding 4-HPR and used as blank.

### 2.4 Determination of the Encapsulation Efficiency and Drug Loading Capacity

The amount of 4-HPR present in the freshly prepared micelles was spectrophotometrically measured after their dilution in methanol, as previously described, and the drug entrapment efficiency percentage (EE%) was subsequently calculated according to the formula in Equation (1) [21].


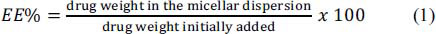


The aliquots of the filtrates were transferred into 5 mL glass vials and stored in a freezer at −20 °C overnight for lyophilization using a freeze–dryer system (ALPHA 1–4 LD plus, Christ Osterode, Germany). The freeze-drying cycle had a duration of 48 h with a condenser temperature of −55 °C and a pressure of 0.021 mbar. The dried samples were stored at -20 °C and reconstituted at room temperature with Milli-Q water to their original concentrations prior to use. The drug loading (DL%) was determined according to the formula in Equation (2) starting from a weighed lyophilized 4-HPR:TPGS:DSPE-PEG powder sample [21]. In addition, for the EE% and DL% calculations, the authors assumed that the amount of free drug in the solution was negligible owing to its poor solubility in water.


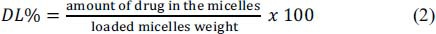


### 2.5 Dynamic Light Scattering Analysis

The mean diameter (Z-average), polydispersity index (PDI), and zeta potential (ƺ) of the micellar dispersions were recorded at 25 °C using a Malvern Nano ZS90 light scattering apparatus (Malvern Instruments Ltd., Worcestershire, UK) at a scattering angle of 90°. The apparent equivalent hydrodynamic *radii* of the micelles were calculated using the Stokes-Einstein equation. For the measurements, aliquots of micellar dispersions were withdrawn and diluted to a count rate > 200 kcps. The results from these experiments were expressed as mean ± SD of three measurements of ten runs per sample.

### 2.6 Differential Scanning Calorimetry (DSC)

Moreover, to confirm the entrapment of 4-HPR, a DSC analysis was performed. The thermal properties of lyophilized 4-HPR:TPGS: DSPE-PEG 1:50:46.6 (w/w), free 4-HPR and TPGS: DSPE-PEG 50:46.6 (w/w), and of the physical mixture of the raw materials in equal ratio to that of the preparative mixture were studied using Discovery SDT 650 equipped with TRIOS software (TA Instrument, New Castle, DE, USA). The instrument was calibrated with sapphire and zinc, and about 4 mg of samples were crimped in alumina pans. The thermograms were recorded from 10 to 200 °C at a heating rate of 10 °C/min under nitrogen flow.

### 2.7 Formulation Stability

#### 2.7.1 Kinetic Stability

The kinetic stability of the micelles was investigated by storing the colloidal dispersions in the liquid state at 4 °C in a fridge and visually observed after 24, 48, and 72 h to detect possible signs of precipitation. At each time point, the drug concentration in the solution was determined after filtration to remove the 4-HPR, which was eventually released. The measurements were made in triplicate, and the results were reported as the mean ± SD.

#### 2.7.2 Stability Upon Dilution

The freshly prepared micellar dispersions were assayed for their stability upon dilution to predict their behaviour after intravenous administration. The samples were diluted to 1:50, 1:100, 1:250, 1:500, and 1:1000 with phosphate buffer saline (PBS, pH = 7.4) containing bovine serum albumin (BSA, 5% w/v) to mimic *in vivo* conditions. After dilution, the samples were stored at 37 °C for 6 h, or 24 h, in incubator WTB © BINDER GmbH 2015-2020 (Im Mittleren Ösch 5, D-78532 Tuttlingen, Germany) and finally filtered with a 0.22 µm filter to spectrophotometrically assess changes in drug concentration. Three independent experiments were performed, and the results were reported as the mean drug loss % ± SD.

### 2.8 *In vitro* Drug Release

The *in vitro* studies of drug released from the 4-HPR:TPGS:DSPE-PEG 1:50:46.6 were carried out as previously reported [29]. Briefly, a weighed amount of 4-HPR:TPGS: DSPE-PEG 1:50:46.6 lyophilized powder (195 mg), equivalent to 3.9 mg drug, was reconstituted with 3mL of PBS. The colloidal suspension was filled in a dialysis tube (CE Dialysis Tubing MW CO 100–500Da, SpectrumTM, Spectra/Pore®, USA), allowing the diffusion only of the free drug. The drug suspension was dialyzed against 20mL of PBS, containing 1-octanol (4:1, v:v), used to extract the drug diffused through the dialysis membrane and ensure sink conditions [30]. The dialysis tube was maintained in the aqueous environment, and aliquots of the solvent were collected and replaced by fresh 1-octanol at fixed time points. The system was thermostated at 37±0.5°C and maintained under stirring at 100 rpm. The concentration of the drug in the releasing compartment was evaluated by a spectrophotometric analysis after dilution with methanol. Each measurement was done in triplicate, and the results were expressed as mean 4-HPR cumulative release percentages (CR %) ± SD, according to Equation (3):


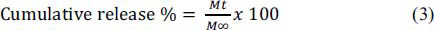


where *M_t_* is the total amount of 4-HPR released in the medium, including the amount being sampled every time point, and *M_∞_* is the initial 4-HPR amount in the dialysis tube [31]. The data obtained from the drug release study were suitable for different release kinetics models like Higuchi, zero-order, first-order, Korsmeyer–Peppas, and Hixson–Crowell. The best-fit model was considered with the highest determination coefficient (*R*^2^) value. The linear regressions and their equations were provided by Microsoft Excel software 365 using the ordinary least square (OLS) method.

### 2.9 Biological Studies

The SK-N-BE-2C and HTLA-230 neuroblastoma cells were maintained in complete medium (Dulbecco’s modified Eagle medium; Sigma) containing 10% v/v heat-inactivated fetal bovine serum (Gibco-Invitrogen S.r.l., Carlsbad, CA, USA) and 50 IU/mL penicillin G; 50 μg/mL streptomycin sulphate; and 2 mM L-glutamine (all reagents from Euroclone S.p.A., Milan, Italy). In order to examine the changes in cell viability, the SK-N-BE-2C cells were seeded in quadruplicate in a 96w plate at the concentration of 1× 10^4^-3 × 10^3^ cells per well in 200 μL of complete medium. After 24 h, the medium was removed, and the cells were exposed for 24 h or 48 h to i) fresh complete medium, ii) different drug concentrations (0.5. 1, 2.5, 5, 10, and 20 μM) either free or entrapped into the mixed micelles or iii) void micelles with the corresponding surfactants concentrations (0.05, 0.10, 0.24, 0.50, 0.99, 1.98 mM). The viability of cells was evaluated by CyQUANT^®^ Direct Cell Proliferation Assay (Thermo Fisher Scientific, Life Technologies, MB, Italy) in according to the manufacturer’s instructions by using the monochromator-based M200 plate reader (Tecan, Männedorf, Switzerland) set at 480/535 nm.

### 2.10 Statistical Analysis

The statistical analyses were determined by the *t*-test with the two-tailed Welch’s correction or by two-way ANOVA with Bonferroni post hoc test, using GraphPad Prism 5 (GraphPad Software v5.0, San Diego, CA, USA). The differences were significant when p-value ranges were: * = *p* < 0.05, ** = *p* < 0.01, *** = *p* < 0.001.

## 3 RESULTS AND DISCUSSION

### 3.1 Surfactants Suitability for 4-HPR Micellization

The selection of TPGS and DSPE-PEG as surfactants suitable to enhance 4-HPR solubility was based on our previous results regarding the development of a hydrogel containing retinoic acid encapsulated into TPGS micelles [22]. However, the use of TPGS as a unique emulsifier is appropriate for topic or oral formulation but not for injectable preparations since it is endowed with a CMC of 0.13 mM. For this reason, DSPE-PEG was selected as a secondary surfactant for its biocompatibility, safety, and high stability of its self-assembled systems [32]. Indeed, DSPE-PEG_5000_ is reported to have 6.4 x 10^-6^ M CMC, and it has already been employed for the encapsulation of paclitaxel, demonstrating low systemic toxicity after intravenous administration. A DSPE-PEG_2000_ micellar preparation showed a CMC value of 1.1 x 10^-5^ M, and mixed micelles made of TPGS/DSPE-PEG_2000_ 1:1 (mol: mol) were of comparable stability [33]. In another work, a molar ratio of 1:3 TPGS: DSPE-PEG_2000_ was used for the successful encapsulation of barberine [34]. While PEG is the most commonly used hydrophilic chain because of its stealth property, the composition of the hydrophobic *core* is strongly related to the chemical structure of the molecules intended to be encapsulated. The presence of TPGS has been recognized to increase drug loading, thanks to the major solubilization ability of the tocopherol moiety [34, 35]. A method to predict the solubility of drugs in tocopherols is the SVE assay. A SVE parameter of at least 10 would indicate an acceptable drug solubility in vitamin E [28]. As expected, 4-HPR showed a greater solubility in chloroform (80.0 ± 2.0 mg/mL) than in methanol (3.6 ± 0.5 mg/mL), and the resulting SVE value was about 22, thus indicating the suitability of the tocopherol residue to solubilize 4-HPR. The molar fraction of TPGS was selected to be two-fold greater than that of DSPE-PEG, hoping to exploit this solubilizing capacity.

Subsequently, mixed micelles made of TPGS and DSPE-PEG (2:1 mol: mol) were prepared using the shake-flask method to investigate the efficiency of the two surfactants in drug micellization. For this purpose, the increasing amounts of the two surfactants were added to a 4-HPR saturated water solution. Each sample was left to equilibrate until the drug concentration in the solution was constant. The presence of the surfactants enhanced 4-HPR apparent solubility, whose concentration at equilibrium was determined using a previously constructed linear calibration curve (Fig. **S1**, in Supplementary Materials). The high value of the determination coefficient (*R*^2^= 0.9999) assured linearity for 4-HPR concentrations in the range of 2.95-4.00 µM. As reported in Fig. (**1A**), at the maximum concentration of surfactants (12.0 mM), the total drug concentration in solution reached the value of 240 µM, thus establishing an improvement by 55-folds (being 4-HPR water solubility equal to 0.004 mM) [26]. (Supplementary Materials).


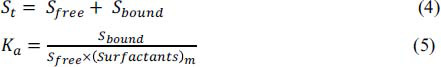


**Fig. (1):**
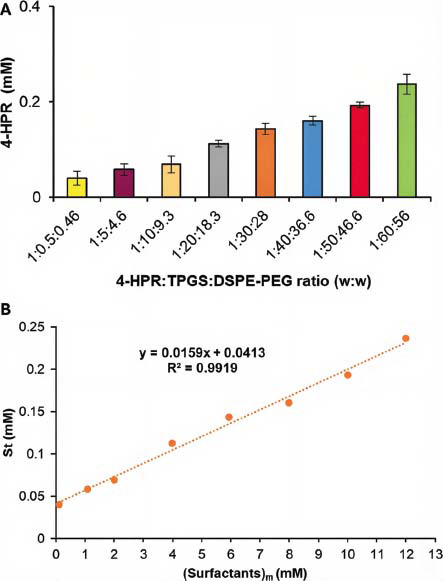
4-HPR apparent solubility as a function of drug:TPGS: DSPE-PEG w/w ratio, measured at 37 °C by the equilibrium method, each value represents the mean ± standard deviation (SD) (*n* = 3) (**A**) Linear regression obtained by plotting drug total solubility (St) versus the concentration of TPGS and DSPE-PEG forming micelles (Surfactants)m (**B**).

As shown in Fig. (**1B**), in the range of weight ratios tested, the surfactants’ concentration and 4-HPR apparent solubility are linearly related, thus suggesting that the micellization took place even at the lowest concentrations and that the system was always above its CMC. The estimation of the equilibrium constant (Ka) for the free form of the drug and the encapsulated drug can be deduced by the following Equations (4), (5) and (6):


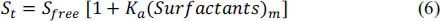


where (Surfactants)_m_ is the concentration of TPGS and DSPE-PEG forming micelles, (S_free_) is the free 4-HPR concentration, (S_t_) is the total 4-HPR concentration, (S_bound_) is the 4-HPR amount encapsulated [25]. The free drug concentration was estimated to be equal to 4-HPR solubility in water. By plotting S_t_ versus (Surfactants)_m_, the linear regression in Fig. (**1B**) was obtained, whose Equation corresponded to Equation 6. Accordingly, a Ka value of 3.64 mM^-1^ was obtained by dividing its slope by S_free_ (Fig. **1B**). This value is higher than that of other previous TPGS-containing formulations loaded with paclitaxel and estradiol (0.86 mM^-1^ and 0.22 mM^-1^, respectively), confirming the suitability of system TPGS/DSPE-PEG as encapsulating agent for 4-HPR [25, 36].

### 3.2 Preparation and Characterization of Loaded TPGS/DSPE-PEG Micelles

The same drug: surfactant ratios, used above in the direct co-dissolution study, were also tested for the preparation of the micelles by the solvent-casting method. In the first method, during heating, the *core* of the structures undergoes dehydration, which leads to the formation of micellar structures [37]. Nevertheless, to enhance encapsulation efficiency, the solvent-casting method represents a valid alternative since after a rotary-evaporation of the solvent under vacuum, the drug molecules remain tightly entangled with the surfactants, thus assuring a higher encapsulation. Here, chloroform was selected as an organic solvent as it was able to solubilize all the components of the mixture. After evaporation of the solvent, a transparent waxy semisolid film formed due to the low TPGS melting point (37-41 °C). When room temperature was achieved, an opalescent and solid film was obtained. Furthermore, to avoid the formation of supersaturated and unstable solutions, the hydration was carried out at room temperature, and in the end, all the formulations appeared yellowish and cloudy, needing filtration to separate the non-encapsulated drug particles. In particular, eight formulations were prepared up to 4-HPR:TPGS: DSPE-PEG 1:60:56 ratio(w/w), and greater increments of surfactant weights were avoided for safety consideration [38]. Furthermore, polymeric nanocarrier materials with surfactant ability may inhibit the activity of cytochromes P450, thus leading to significant changes in exposure to certain drugs [39]. As shown in Fig. (**2**), the total 4-HPR solubility increased almost proportionally with surfactants’ increment up to a 1:50:46.6 ratio. Notably, the drug concentrations ranged from 0.13±0.02 to 1.45±0.13 mM, with the latter corresponding to a 363-fold increase in 4-HPR aqueous solubility. Plain micelles made of TPGS alone were also prepared in the ratio of 1:50 and 1:60 4-HPR: TPGS (w/w) to explore the effect of TPGS in the micellization of the drug. These formulations provided an enhancement of drug total solubility superior to the corresponding mixed micelles (7.42 ± 0.89 and 8.64 ± 1.47 mM for 1:50 and 1:60 4-HPR: TPGS, respectively) however they underwent in a few hours to a massive drug precipitation. These findings suggested that TPGS promotes drug entrapment within the micellar system but also provides supersaturated drug solutions prone to instability. On the other hand, DSPE-PEG alone can not maintain an adequate large hydrophobic core to accommodate a suitable drug amount, as it was proven for paclitaxel [33], thus, the presence of both surfactants is mandatory. Although our solubility increment was lower than that of other synthesized amphiphilic polymers [40, 41], here the micellization occurred by the recruitment of highly biocompatible excipients. TPGS and DSPE-PEG have been approved by FDA as a safe pharmaceutical adjuvant, and TPGS is included in the United States Pharmacopeia, while DSPE-PEG has received regulatory approval in three formulations of doxorubicin (Doxil, LipoDox, and Thermodox), which makes the current micellar delivery system highly translatable to the clinic [38].

For these formulations, the total quantity of the solubilized 4-HPR per mL of the micelle colloidal suspension linearly depended on the concentration of the surfactants in the mixture (Fig. (**2B**); y = 0.0171x + 0.0218 *R*^2^ = 0.9866). This solubilization profile fits well those generally reported for water-insoluble drugs solubilized by micellar systems [42], thus allowing to obtain loaded micelles using still reasonable surfactants concentrations.

Since a good EE% was achieved, the data reported in Table **1** confirmed the suitability of the preparative method adopted. The drug’s positioning inside the micellar *core* is strictly affected by the hydrophobic interactions between the drug and the lipophilic moieties of the surfactants. The hydrophobic interactions represent the driving force in the self-assembling process of the supramolecular systems since they are relatively stronger than Van der Waals interactions or hydrogen bonds [43]. 4-HPR is a molecule with a log P of 7.4. In an aqueous solution, its hydrophobicity can easily drive drug molecules to assemble into the anhydrous *core* of the amphiphiles. The mean DL% was found to be around 3%, as reported in the literature, as most micelle systems suffer from a low drug loading capacity, and different options for overcoming this limit have been proposed [44, 45]. Since we have selected highly biocompatible surfactants, the need for a larger volume and potentially more excipients to be administered should not cause the onset of unwanted toxic effects. Regarding the size distribution, the mean diameters were found to be 12 nm with a mean PDI of 0.19, as reported in Table **1** and in Fig. (**S2**). However, the formulation at the lowest ratio of ingredients showed a significant difference both in size distribution and Zeta potential (Fig. **S2B** and **S3B**). Indeed, for this formulation, there was a one-dimensional family at about 12 nm attributable to micelles and another at 326 nm. The latter could be due to an inefficient separation by filtration of the raw 4-HPR powder since the amount of unencapsulated drug particles was massive for this surfactant:drug ratio. Overall, these data were in agreement with other studies based on mixed micelles made of TPGS and DSPE-PEG at 1:1 and 1:3 (mol:mol) ratios [33, 34]. Notably, when the ratio was 1:1, the mean diameter was around 14 nm, while in the latter case, a shift toward greater mean diameters (26 nm) was observed, therefore, we can hypothesize that vitamin E strongly influences the tight packing of the micellar *core*. All the prepared formulations showed negative Zeta potentials, whose values were determined by the negative charge of the DSPE-PEG, being TPGS, a non-ionizable neutral molecule [34]. Only the formulation 1:0.5:0.46 HPR:TPGS: DSPE-PEG showed a significantly different value, but as aforementioned, this formulation was not completely separated by the unentrapped drug particles. The small sizes and negative surface achieved may suggest that these micelles could be suitable for intravenous administration.

### 3.3 Thermal Analysis

The drug entrapped inside the micellar structures may be found as molecularly dispersed or in crystallized form. To this end, the thermal profile of the 4-HPR:TPGS: DSPE-PEG 1:50:46.6 lyophilized formulation was compared to the corresponding physical mixture, as well as to pure 4-HPR and surfactants. As depicted in the thermograms reported in Fig. (**3**), 4-HPR showed a sharp peak at 174 °C associated with its melting point. Lyophilized TPGS DSPE-PEG (void micelles) gave an endothermic peak of melting at around 43 °C.

The DSC thermograms of the lyophilized 4-HPR formulation showed only the melting point of the mixture TPGS/DSPE-PEG. However, also the physical mixture profile evidenced only the melting point of TPGS/DSPE-PEG with the disappearance of the peak at 174 °C due to drug *in situ* solubilization in the melted waxy TPGS during DSC measurement. Therefore, the melting endotherm peak of 4-HPR of the physical mixture, prepared with the same drug surfactant ratio as that of 4-HPR loaded micelles, could not be detected. This phenomenon was also observed for a physical mixture made of paclitaxel and DSPE-PEG_5000_, where the disappearance of the drug melting peak occurred similarly [32] .

### 3.4 Stability of Loaded Mixed Micelles

For micelles, stability is generally defined in terms of thermodynamic as well as kinetic stability. Thermodynamic stability is achieved when the surfactant concentration is above the CMC. However, micelles are a dynamic system, and the exchange rate of amphiphilic unimers between the bulk and the supramolecular system may determine its disassembling and opening. On the basis of our experience, when the hydration of the thin film occurs at room temperature, the stability of the self-assembled structures is greater than when the hydration is performed under light heating, as is reported in some works [33, 46]. In this case, when the hydration was performed at 40 °C, less cloudy colloidal suspensions were obtained, but only after a few hours precipitation of the drug occurred, thus suggesting the establishment of supersaturated drug solutions. For this reason, we opted for a long hydration at room temperature, which led to a minor drug solubilization but hopefully to greater stability of the systems. In addition, to determine if our self-assembled nanostructures underwent some changes in their composition at selected time points, the drug leakage was detected. As shown in Fig. (**4**), in the presence of high amounts of surfactants, the drug concentrations were almost constant, with a slight decrease at 72 h storage. On the contrary, the formulations with ratios below 1:40:36.6 showed evident drug leakage already after 24 h storage. Notably, in Table **1**, the drug loss % was reported for each formulation.

*In vivo,* the stability of micelles mainly depends on the CMC values. When a micellar colloidal dispersion is diluted in the systemic circulation to a very low concentration, the surfactant may not be able to retain the self-assembled structures. Moreover, to mimic the *in vivo* conditions to whom our 4-HPR formulation could be exposed after intravenous administration, the micellar dispersions were diluted with PBS containing 5% BSA, 5%, stored at 37 °C and finally filtered to spectrophotometrically assess changes in drug concentration in solution. The effect of the dilution could not be monitored by investigating changes in size distribution since the kcps of the most diluted samples were too low to perform a reliable analysis, and BSA showed sizes overlapping those of the micelles. However, if the opening of the micelles occurs, the decrease in drug content in solution due to 4-HPR insolubility may be indicative of the thermodynamic stability of the formulations. As shown in Fig. (**5**), the 1:0.5:0.46 formulation resulted in inconsistency at every degree of dilution, while the 1:5:4.6 formulation started changing deeply at 1 to 250 dilution. The other samples suffered a loss of drug percentage of less than 16% after 6 h and 20% after 24 h, proving a good endurance to dilution. Since thermodynamic stability depends on the CMC value, in Table **2**, the concentration of surfactants at each dilution was reported. Comparing the molar concentrations of the micelles’ forming material with the drug loss % data, we can affirm that the systems were stable when the concentration of the surfactants was at least 2.97E-05 M, deducing that the CMC value of these nanostructures could correspond to this order of magnitude. This value indicates that these mixed micelles are more resistant to dilution than micelles made only of TPGS, having a CMC of 0.13 mM [47], and proves that they have a stability comparable to other TPGS/DSPE-PEG mixed micelles (CMC = 1.1 x 10^-5^ M), even if containing a different molar ratio between the two surfactants [33].

### 3.5 *In vitro* Release Kinetic

**Fig. (6):**
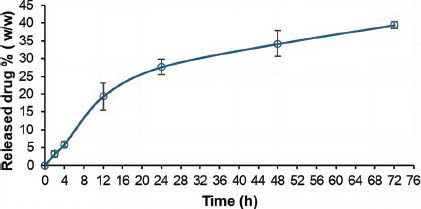
Cumulative release (%) of 4-HPR at 37 °C and pH = 7.4 over time. Each value represents the mean ± standard deviation (SD) (*n* = 3).

Release studies were performed from a re-dispersed lyophilized powder prepared from the blend 1:50:46.6 4-HPR:TPGS: DSPE-PEG. The *in vitro* release of 4-HPR from mixed micelles was quite slow, with no more than 28% of the drug being released after 24 h, suggesting that the drug incorporated tended to remain firmly inside the micelles (Fig. **6**). The release rate strongly affected by the drug polarity and nature of the lipophilic moieties. Typically, lipophilic drugs remain completely sequestered inside the *core,* and the rate of drug diffusion through the micelles depends mainly on the water permeability of the *core* to the aqueous media. In these mixed micelles, TPGS, with its phytyl side chain, is expected to increase hydrophobicity and strengthen the hydrophobic interactions with respect to plain micelles made of DSPE-PEG. The experimental data were fitted with the most common mathematical kinetic models to investigate the main kinetics and physicochemical mechanisms governing the 4-HPR release (Fig. **S4**-**S8**, Table **1S**) [48].

The R^2^ values showed that the Korsmeyer-Peppas model best fits the 4-HPR release profile (R^2^ = 0.9783). Generally, for hydrophobic drugs located in the micellar core, release can follow two major pathways *i.e*., the slow dissociation of the supramolecular structure and diffusion of the payload. Concerning the release profiles fitting the Korsmeyer Peppas model, the main mechanisms could be diffusional or more complex processes (Case II or Super Case II transport) depending on the value of the diffusional or transport exponent n ^KP^ where n corresponds to the slope of the equation in Fig. (**S8**) [49]. Particularly, when n ≤ 0.45, the drug release mainly follows Fickian diffusion mechanisms, when 0.45 < n < 0.89, it corresponds to a non-Fickian transport, if n = 0.89 to a Case II transport, and finally, when n > 0.89 to a super case II transport as in our case, being n = 9.6. According to these results, the release of 4-HPR from the TPGS-based formulation was governed by very complex mechanisms that changed over time.

### 3.6 Antitumor Activity Against Neuroblastoma Cells

**Fig. (7):**
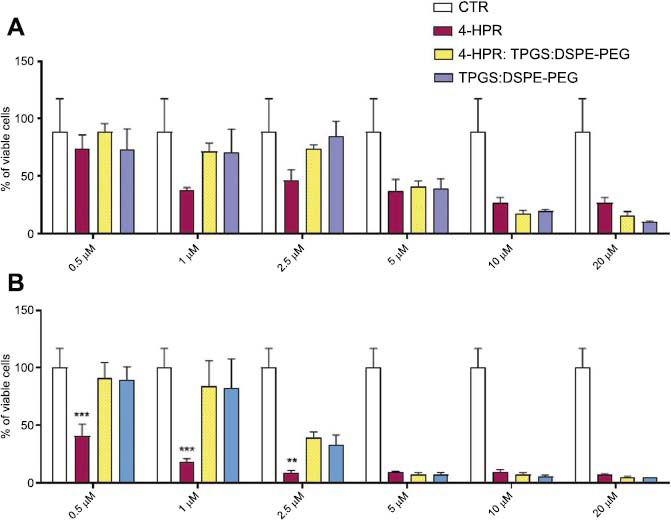
Viability of human neuroblastoma HTLA-230 cells treated with increasing concentrations of 4-HPR as reported on the x-axis, either in the free form or encapsulated into 4-HPR:TPGS: DSPE-PEG 1:50 (w/w) formulation, or with TPGS: DSPE-PEG (2:1 mol/mol, void micelles) at 50, 100, 240, 500, 990 and 1,980 µM concentrations after 24 h (**A**) and 48 h (**B**) of exposure. The experiments were carried out three times, and the data are represented as the average ± S.D (*n* = 5). *p<0.05, **p<0.01, ***p<0.001 *vs.* 4-HPR:TPGS:DSPE-PEG 1:50:46.6 (w/w) formulation.

**Fig. (8):**
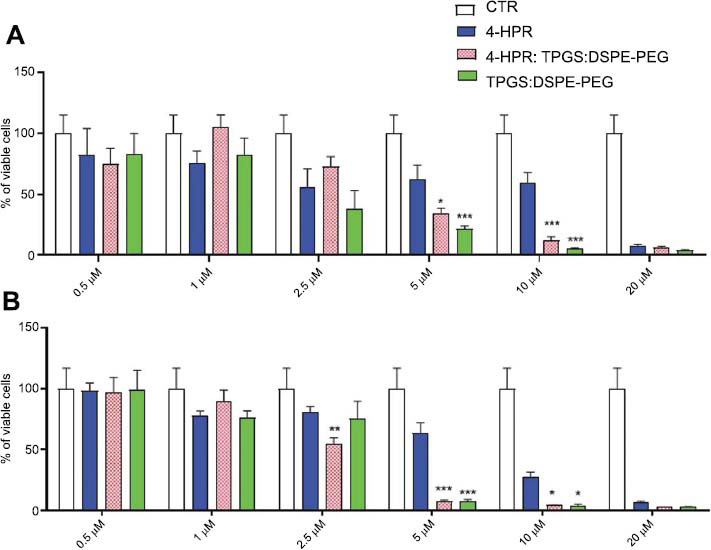
Viability of human neuroblastoma SK-N-BE-2C cells treated with increasing concentrations of 4-HPR as reported on the x-axis, either in the free form or encapsulated into 4-HPR:TPGS: DSPE-PEG 1:50:46.6 (w/w) formulation, or with TPGS: DSPE-PEG (2:1 mol/mol, void micelles) at 50, 100, 240, 500, 990 and 1,980 µM concentrations after 24 h (**A**) and 48 h (**B**) of exposure. The experiments were carried out three times, and data are represented as the average ± S.D (*n* = 5). *p<0.05, **p<0.01, ***p<0.001 *vs.* 4-HPR.

The formulation 1:50:46.6 4-HPR:TPGS: DSPE-PEG was used to test the cytotoxic activity of the micellar complexes against neuroblastoma cell lines. The cells were exposed to drug concentrations ranging from 0.5 to 20.0 µM, either free or entrapped in the micellar system (Figs. **7** and **8**). The cells were also treated with the equivalent amounts of micelles’ forming material ranging from 0.05 to 1.98 mM and corresponding to the concentrations present in the loaded micelles. The two lines were selected to be typically one more sensitive (HTLA-230) and the other more resistant (SK-N-BE-2C) to drugs.

Furthermore, to better compare the anticancer effects of all samples, their IC50 values were determined (Table **3**).

In the case of sensitive HTLA-230 cells, after 24 h of exposure, the cytotoxic activity of free 4-HPR, micellar 4-HPR, and void micelles do not differ significantly at all concentrations tested. As evidenced by data reported in Table **3**, the IC50 values were 2.05 µM (4-HPR) and 3.42 µM (micellar 4-HPR). Differently, after 48 h of exposure, for concentrations ≤ 2.50 µM, the free form of the drug resulted significantly more effective than the micellar 4-HPR (Fig. **7B**). Anyway, for concentrations ≥ 5.00 µM, the anticancer effects of all samples were comparable and not significantly dissimilar. More promising results were obtained on the most resistant SK-N-BE-2C cells. As evidenced in Fig. (**8**), micellar 4-HPR was significantly more effective than free 4-HPR with IC50 values 1.7 and 2.4-fold more active than free 4-HPR after 24 and 48 h, respectively. Concerning void micelles, the results obtained here were in good agreement with those reported earlier [18, 50]. Particularly, TPGS was tested on different cancer cell lines, showing the strongest effect on MCF-7-ADR, with an IC50 value of 102 µM at 24h, and the weakest on HeLa with an IC50 value of 251 µM at 24h. These results were comparable with our data since considering that the blend TPGS: DSPE-PEG was at a molar ratio of 2:1, the IC50 value of TPGS on SK-N-BE-2C was 124 µM at 24 h. Our results on SK-N-BE-2C confirmed that the TPGS system may act as a chemosensitizer and potentiate the cytotoxic effects of 4-HPR. The loaded micelles could exert pleiotropic effects by activating different pathways and thus contribute more efficiently to the decrease in the viability of the most resistant cell line. More in-depth studies aiming at clarifying the cellular mechanisms on the basis of these effects will be the focus of future investigations.

## CONCLUSION

Exploring established and FDA-approved excipients offers the advantage of easily translating research studies into clinical trials. Here, TPGS/DSPE-PEG mixed micelles were tested for their ability to encapsulate the lipophilic drug 4-HPR. TPGS was selected for its solubilization ability and capacity to fortify loaded micellar structures by triggering strong hydrophobic interactions, while DSPE-PEG was for its biocompatibility and propensity of forming micelles with low CMC. 4-HPR yielded some encouraging results in clinical studies, but more efforts are needed to increase drug bioavailability. To this aim, mixed micelles made of TPGS and DSPE-PEG containing 4-HPR were successfully prepared with a mean diameter of 12 nm, negative Zeta potential, and a narrow dimensional distribution, leading to an increment of the drug's apparent solubility of 363-fold. The loaded delivery system was able to provide a slow cargo release (28% after 24 h) and a cytotoxic activity significantly superior to free 4-HPR on the most resistant neuroblastoma cells (2.4-fold more active than free 4-HPR after 48 h). In conclusion, this formulation may be useful for the development of more effective and less toxic anticancer treatments.

## Figures and Tables

**Fig. (2):**
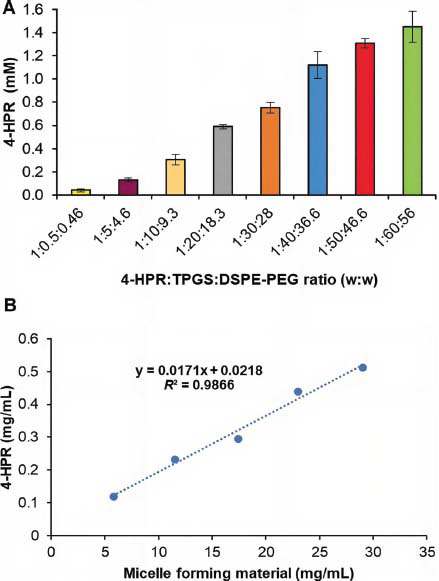
Increment of 4-HPR total solubility as a function of 4-HPR:TPGS: DSPE-PEG (w/w) ratio present in the preparative mixture by solvent-casting method, each value represents the mean ± standard deviation (SD) (*n* = 3) (**A**); effect of the total micelle-forming material concentration on 4-HPR concentration in micellar suspension (**B**).

**Fig. (3):**
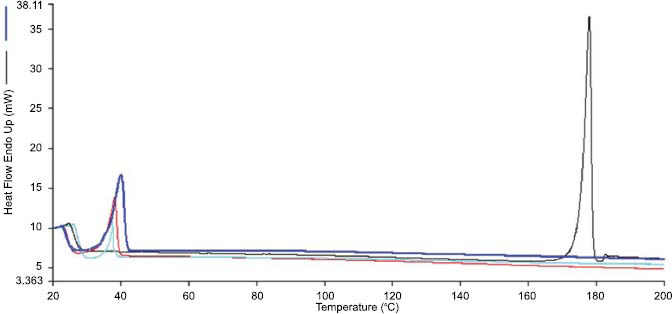
Thermograms of the selected lyophilized 4-HPR-TPGS: DSPE-PEG 1:50:46.6 formulation (light blue line), lyophilized TPGS: DSPE-PEG (red line), 4-HPR-TPGS: DSPE-PEG physical mixture (blue line) and 4-HPR (black line).

**Fig. (4):**
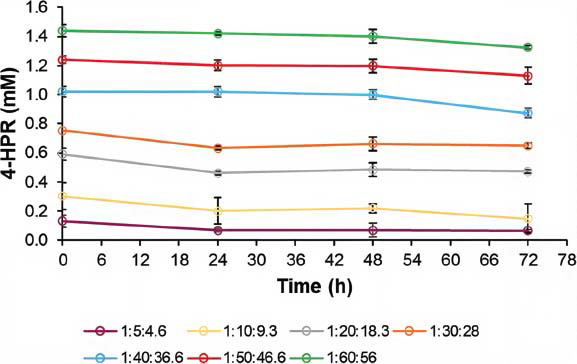
Stability over time of aqueous micellar dispersions at 1:5:4.6, 1:10:9.3, 1:20:18.3, 1:30:28, 1:40:36.6, 1:50:46.6, and 1:60:56 4-HPR:TPGS: DSPE-PEG (w/w) ratio maintained at 4°C. Each value represents the mean ± standard deviation (SD) (*n* = 3).

**Fig. (5):**
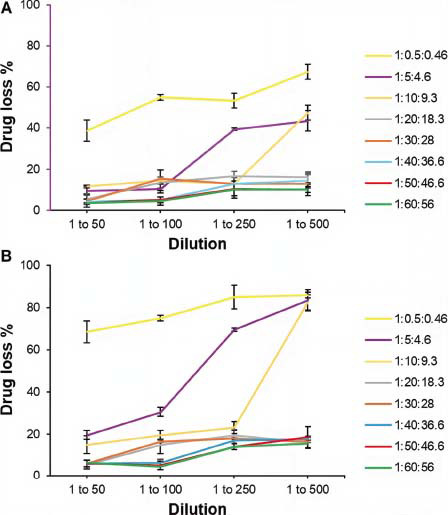
Drug loss % as a function of dilution with PBS (pH = 7.4) containing bovine serum albumin (BSA, 5% w/v) at 37 °C after 6 h (**A**) and 24 h (**B**). Each value represents the mean ± standard deviation (SD) (*n* = 3).

**Table 1:** Physicochemical properties and drug loss % after 72 h storage at 4°C of different 4-HPR micellar preparations. Drug Loading (DL), Encapsulation Efficiency (EE). Each value represents the mean ± standard deviation (SD) (*n* = 3).

**Sample**	**4-HPR Total Solubility (mM)**	**EE%**	**DL%**	**Size (nm)**	**PDI**	**ʒ (mV)**	**Drug Loss %**
1:0.5:0.46	0.04±0.01	2±1	NT	15.7±2.6	0.46±0.03	-4±4	NT
1:5:4.6	0.13±0.02	7±2	NT	11.8±3.1	0.21±0.06	-14±3	54±6
1:10:9.3	0.30±0.04	17±4	1.3±0.2	11.8±0.4	0.17±0.03	-12±2	50±4
1:20:18.3	0.59±0.02	33±2	2.7±0.1	12.7±1.4	0.14±0.02	-11±2	20±2
1:30:28	0.75±0.05	43±3	2.9±0.2	11.4±1.8	0.12±0.06	-13±3	14±5
1:40:36.6	1.12±0.11	64±6	3.1±0.3	11.7±2.6	0.14±0.01	-13±6	14±3
1:50:46.6	1.31±0.04	70±4	3.4±0.2	11.6±3.1	0.13±0.02	-12±4	9±4
1:60:56	1.45±0.13	88±8	3.6±0.5	12.1±1.3	0.17±0.04	-10±2	8±3

**Note:** NT = not determined.

**Table 2:** Surfactants’ concentration after dilution with PBS (pH = 7.4) containing bovine serum albumin (BSA, 5% w/v). Each value represents the mean ± standard deviation (SD) (*n* = 3).

**4-HPR:TPGS:DSPE-PEG (w:w) Ratio**	**Surfactant Concentrations (M)**			
	**1 to 50**	**1 to 100**	**1 to 250**	**1 to 500**
1:0.5:0.46	2.97E-06	1.48E-06	5.93E-07	2.97E-07
1:5:4.6	2.97E-05	1.48E-05	5.93E-06	2.97E-06
1:10:9.3	6.10E-05	3.05 E-05	1.22E-05	6.10E-06
1:20:18.3	0.12E-03	5.92E-05	2.37E-05	1.18E-05
1:30:28	0.18E-03	8.94E-05	3.58E-05	1.79E-05
1:40:36.6	0.24E-03	0.12E-03	4.74E-05	2.37E-05
1:50:46.6	0.30E-03	0.15E-03	5.96E-05	2.98E-05
1:60:56	0.36E-03	0.18E-03	7.15E-05	3.58E-05

**Table 3:** Average IC50 values (µM) ± SD of all samples after 24 and 48 h of exposure to free 4-HPR, encapsulated 4-HPR, or void micelles.

**Exposure time (h)**	**Sample**	**HTLA-230**	**SK-N-BE-2C**
24	4-HPR	2.05±0.60	6.15±1.05
	4-HPR: TPGS: DSPE-PEG	3.42±0.57	3.59±0.25
	TPGS: DSPE-PEG	318.30±12.90	197.40±27.90
48	4-HPR	0.31±0.12	5.46±1.43
	4-HPR:TPGS:DSPE-PEG	1.86±0.58	2.30±0.54
	TPGS:DSPE-PEG	171.1±19.7	254.9±25.6

## Data Availability

The data are available on request to the corresponding author.

## LIST OF ABBREVIATIONS

CMC: Critical Micellle Concentration
DL%: Drug Loading Percentage
DSPE-PEG: 1,2-distearoyl[1]glycero-3-phosphoethanolamine-N- [methoxy(polyethyleneglycol)-2000]
EE%: Entrapment Efficiency Percentage
4-HPR: N-(4- hydroxyphenyl)-retinamide
HBS: HEPES-buffered Saline
P-gp: P-glycoprotein
PEG: Polyethylene Glycol
SVE: Solubility Vitamin E Parameter
TPGS: D-α-tocopheryl Polyethylene Glycol Succinate
