# Supplementary material for: Development of Mixed Micelles for Enhancing Fenretinide Apparent Solubility and Anticancer Activity Against Neuroblastoma Cells
Source: Curr Drug Deliv. 2024 Sep 3;22(7):1017–29. doi: 10.2174/0115672018333862240830072536 (PMC12606610; doi:10.2174/0115672018333862240830072536)
Supplement: Supplementary file 1 [file CDD-22-7-1017_SD1.pdf]

SUPPLEMENTARY MATERIAL

Development of Mixed Micelles for Enhancing Fenretinide Apparent Solubility and Anticancer Activity Against Neuroblastoma Cells

Guendalina Zuccari[1,*], Alessia Zorzoli[2], Danilo Marimpietri[2] and and Silvana Alfei[1]

[1]*Department of Pharmacy, University of Genoa, Viale Benedetto XV 16132 Genoa, Italy;* [2]*Stem Cell Laboratory and Cell Therapy Center, IRCCS Istituto Giannina Gaslini, via Gerolamo Gaslini 5, 16147 Genoa, Italy*

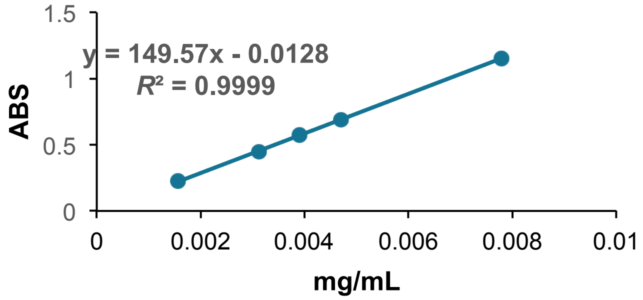

Fig. (S1). 4-HPR calibration curve in methanol at λmax = 364 nm using an UV-Vis spectrophotometer.

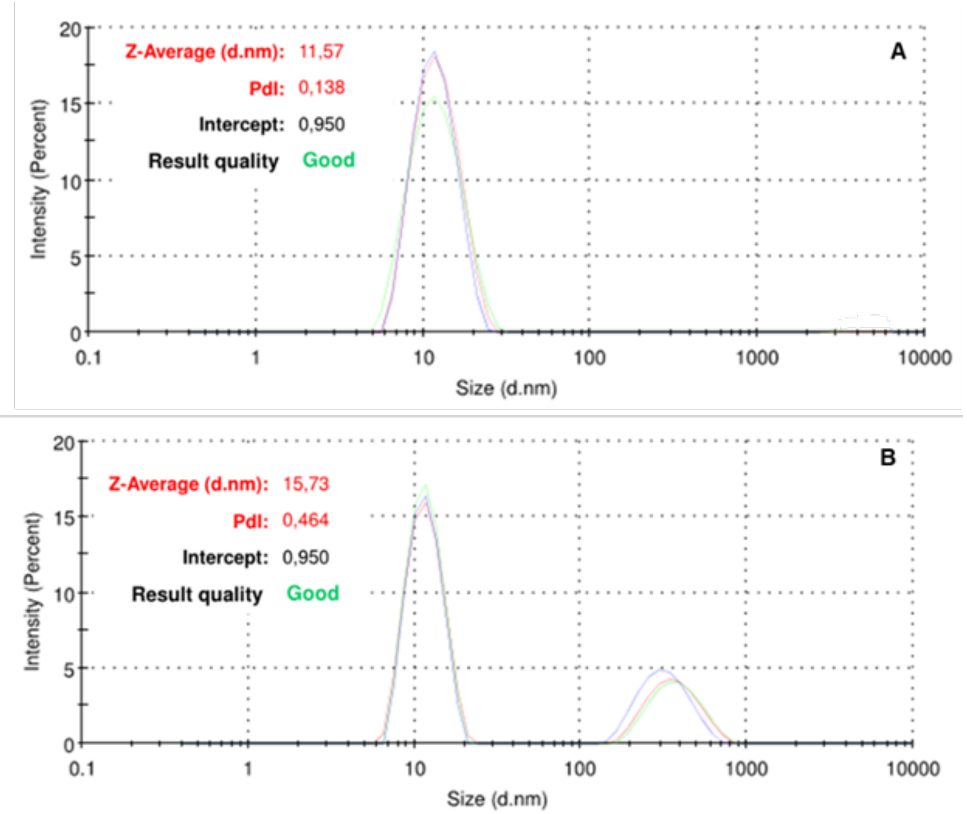

Fig. (S2). Representative size distributions of formulations obtained from 1:50:46.6 (A) and 1:0.5:0.46 (B) 4-HPR:TPGS:DSPE-PEG (w:w) ratio in the preparative mixture. In Fig. S2B two dimensional families are observable: peak 1 (mean diameter = 12 nm) associated to loaded micelles and peak 2 (mean diameter = 326 nm) associated to the presence of drug aggregates due to the low concentration of surfactants.

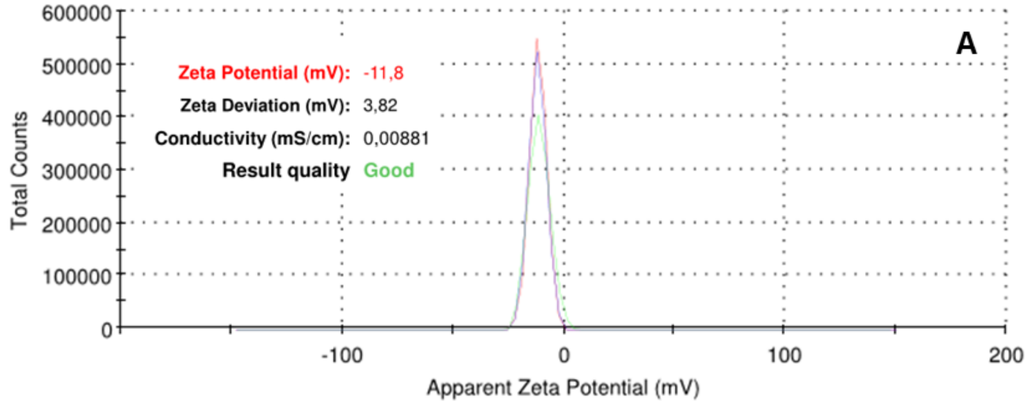

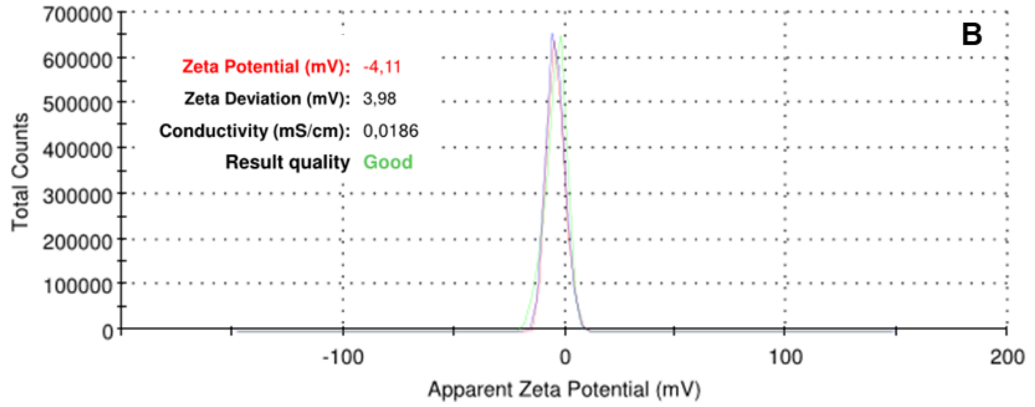

Fig. (S3). Representative distributions of the Zeta potential of formulations obtained from 1:50:46.6 (A) and 1:0.5:0.46 (B) 4-HPR:TPGS:DSPE-PEG (w:w) ratio in the preparative mixture. Fig. S3B shows that the formulation with a lower surfactant concentration has less negative Zeta potential.

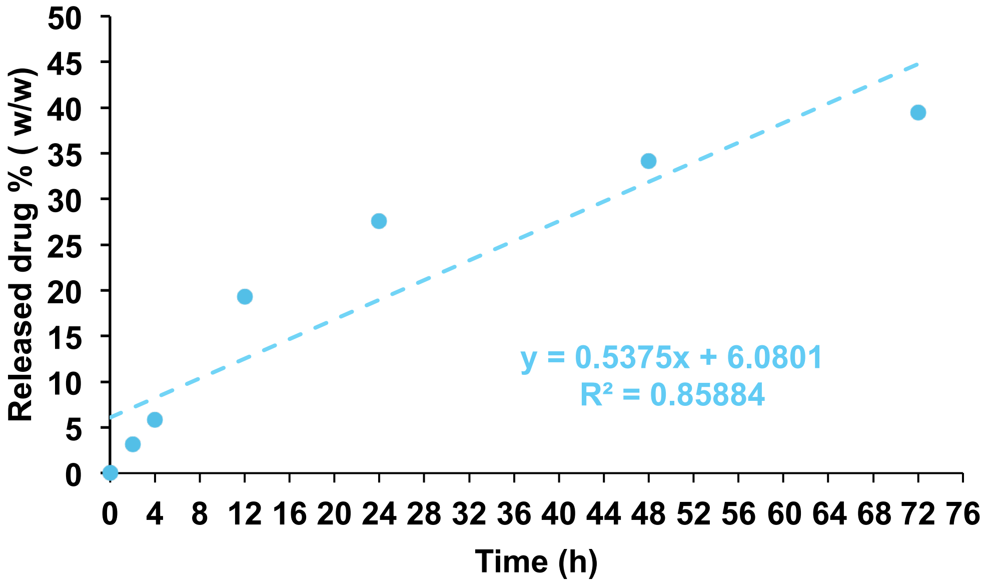

Fig. (S4). Zero order kinetic model.

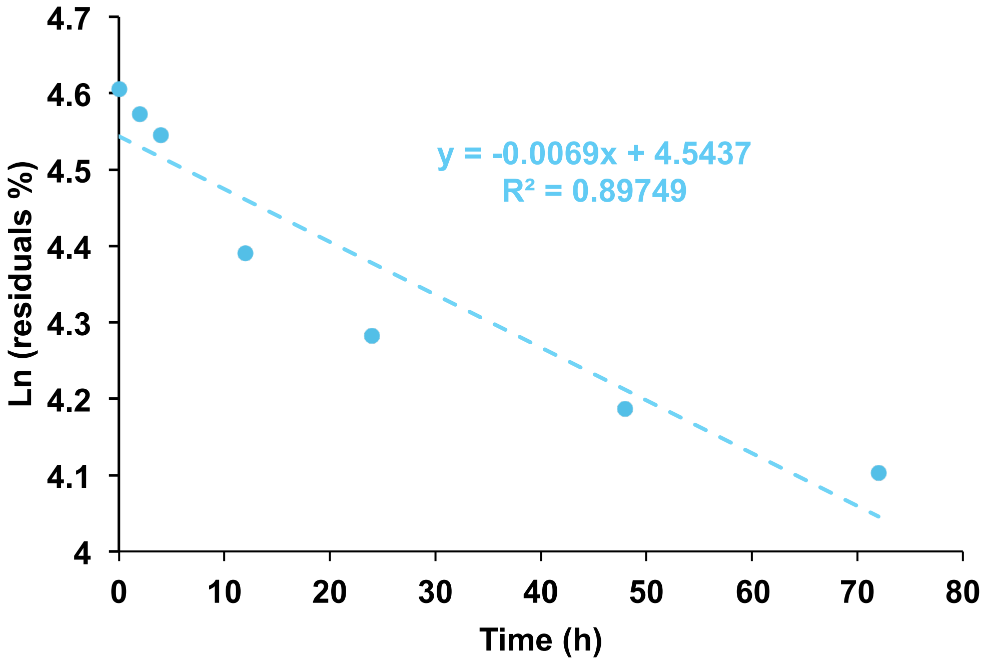

Fig. (S5). First order kinetic model.

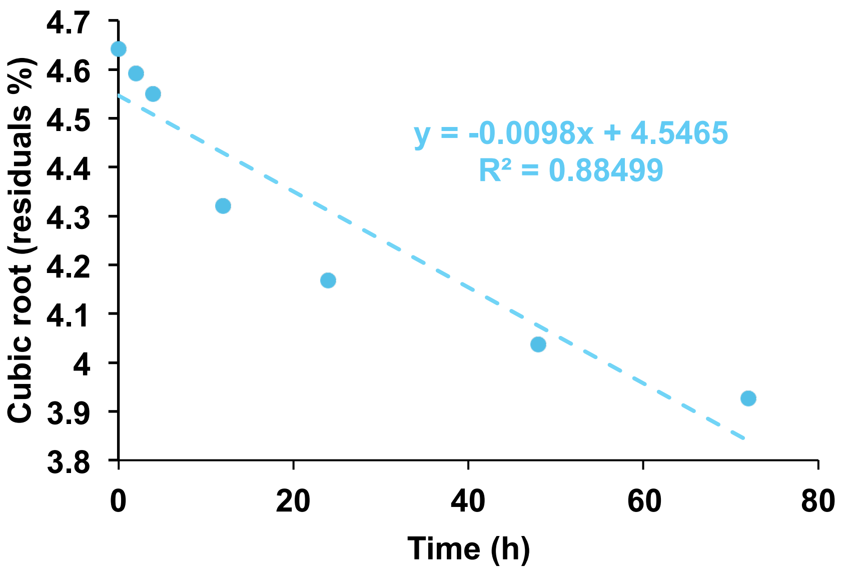

Fig. (S6). Hixson Crowell kinetic model.

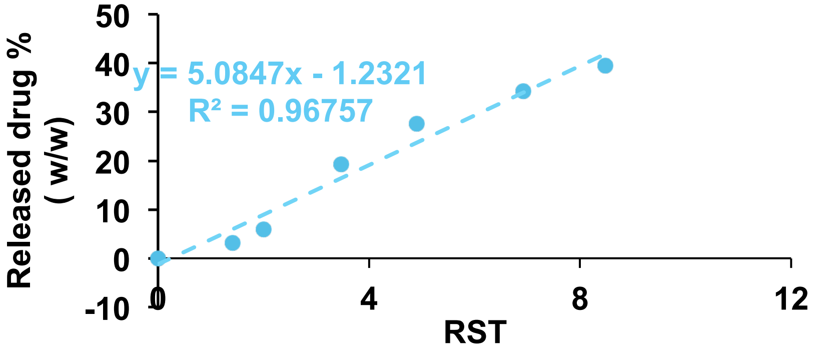

Fig. (S7). Higuchi kinetic model (RST = root square of times).

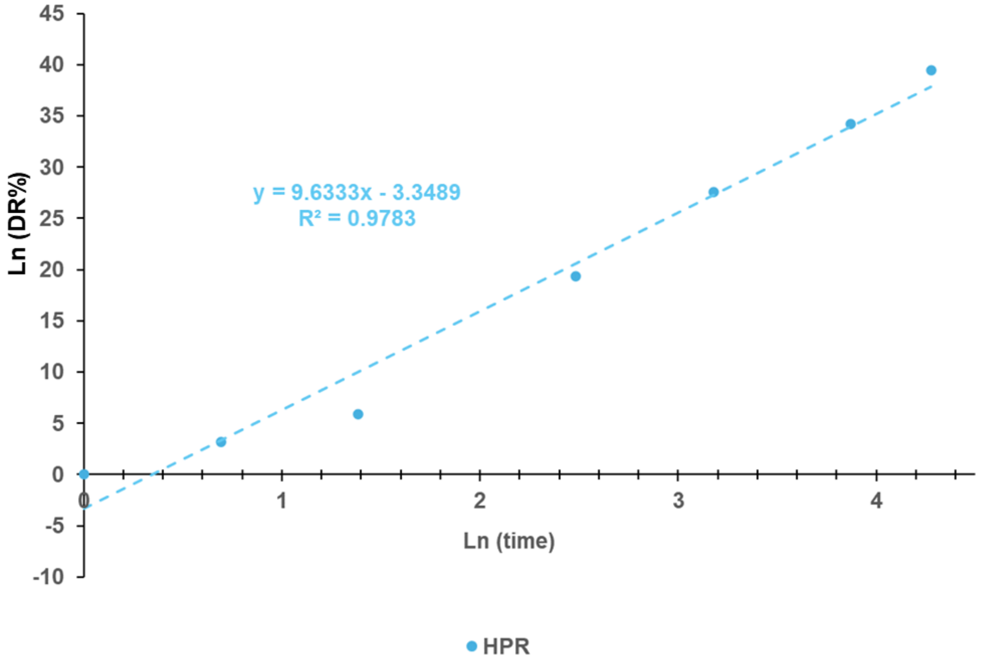

Fig. (S8). Korsmeyer Peppas kinetic model.

Table 1S. Variables to be reported on x and y axes to obtain the dispersion graphs of mathematical models considered in this study, the equations expressing them and R^2 values obtained for all kinetic models tested.

Model	X Axis	Y Axis	Equation	R^2
Zero-order	Times	DR%(t)	$DR\%(t) = K_0 \times t + DR\%(t_0)$	0.8588
First-order	Times	Ln (residual%)	$Ln[R\%(t)] = [R\%(t_0)] + K_1/2.303 \times t$	0.8975
Higuchi	SRT *	DR% (t)	$DR\%(t) = K_H \times t^{1/2}$	0.9676
Korsmeyer-Peppas	Ln (times)	Ln (DR%)	$Ln\ [DR\%(t)] = n_{KP} \times Ln\ (t) + Ln\ (K_{KP})$	0.9783
Hixson-Crowell	Times	Cubic root (residual%)	$\sqrt[3]{1 - F\%(t)} = 1 - K_{HC} \times t$	0.8850

* square root of times; DR% (t) is the drug released percentage at time t; DR% (t_0) is the drug released percentage at the beginning ($t = t_0$); K_0 is the zero-order constant; K_1 is the first order constant; K_{KP} is the transport constant of the Korsmeyer-Peppas model; n_{KP} (also called diffusional or transport exponent) provides information on the possible mechanism(s) governing the process under study; F% (t) is the cumulative fraction percentage of drug released at time t; K_{HC} is the Hixson-Crowell constant; K_H is the Higuchi kinetic constant.
